# Prevalence and Counseling Practices of Contraceptive Use Among Women With Systemic Lupus Erythematosus at a Tertiary Center in Saudi Arabia

**DOI:** 10.7759/cureus.104827

**Published:** 2026-03-07

**Authors:** Abdulaziz A Aljohani, Sydirfan Karim, Abdullah Alzahrani, Abdulrahman Hisn Alduhayyim, Rakan Alghonaim, Maha Abdullah Aljohani, Sulaiman F Alzomia

**Affiliations:** 1 Department of Family and Community Medicine, King Saud University, Riyadh, SAU; 2 College of Medicine, King Saud University, Riyadh, SAU

**Keywords:** contraceptive counseling, reproductive health, saudi arabia, systemic lupus erythematosus, teratogenic medications

## Abstract

Systemic lupus erythematosus (SLE) is a chronic autoimmune disorder that mainly affects women of reproductive age. Management often involves immunosuppressive therapies, such as azathioprine and cyclophosphamide, which carry teratogenic risks. Effective contraceptive counseling is therefore essential, yet data on counseling provision and contraceptive use among women with SLE in Saudi Arabia are limited.

Objective: This study aimed to evaluate the prevalence, patterns, and counseling practices of contraceptive use among women with SLE who were prescribed potentially teratogenic medications at a tertiary center in Riyadh, Saudi Arabia. Additionally, the study examined associations between contraceptive use patterns and key demographic and clinical factors, including educational level, parity, disease duration, and pregnancy risk status.

Methods: This cross-sectional study collected data from electronic health records and conducted phone interviews with women aged 18-45 years with SLE who were receiving azathioprine or cyclophosphamide. Demographics, disease characteristics, contraceptive counseling, and usage patterns were analyzed using descriptive and inferential statistics. Participants were recruited from the outpatient immunology clinics of tertiary hospitals in the central region of Riyadh, Saudi Arabia.

Results: Of the 399 women included in the study, 209 (54.6%) were considered at risk of becoming pregnant. Only 17.2% reported consistent contraceptive use in the past three months, while 64.2% reported intermittent use. Notably, 90.1% of non-users were still prescribed teratogenic medications. Intrauterine devices were the most common method of contraception (38.7%), followed by barrier methods (17.3%). Although 55.3% received contraceptive counseling at the time of diagnosis, less than 40% reported receiving counseling in the past year. Although educational level was found to significantly influence contraceptive behaviors, most non-users were college-educated women. Disease duration was associated with increased use of barrier methods.

Conclusion: The findings reveal that, despite high teratogenic exposure, many Saudi women with SLE lack consistent contraceptive use and ongoing counseling. Compounding this issue, many participants reported an absence of structured, ongoing contraceptive counseling within their care pathways. These findings underscore an urgent need to integrate routine, protocol-driven reproductive health services into standard lupus care. Tailored interventions, including repeated counseling at key disease junctures and accessible contraceptive services, are essential to mitigate the risk of unplanned pregnancies, optimize maternal and fetal outcomes, and address a significant gap in the comprehensive care of this vulnerable population.

## Introduction

Systemic lupus erythematosus (SLE) is a chronic, multisystem autoimmune disease characterized by dysregulated immune responses, leading to inflammation and damage to various organs, including the brain, lungs, kidneys, joints, skin, and blood vessels. SLE, the most prevalent form of lupus, predominantly affects women of reproductive age, with a female-to-male ratio of 9:1. Nevertheless, the risk decreases after menopause [1,2]. In addition to medical intervention, lifestyle modifications can enhance a patient's quality of life and help control the disease [3,4].

Despite the unpredictable course of this disease, pharmacologic interventions have significantly improved patient outcomes. Unfortunately, many of these interventions, such as azathioprine, cyclophosphamide, methotrexate, rituximab, and mycophenolate mofetil, have potentially teratogenic effects, resulting in unsafe pregnancies. Moreover, women with SLE already have an increased risk of pregnancy complications, such as early miscarriages, ectopic pregnancies, late miscarriages or stillbirths, and induced abortions. Therefore, reproductive health considerations are paramount for these women, particularly those prescribed teratogenic drugs. Using effective contraception can significantly reduce unplanned pregnancies and lead to better health outcomes [5-7]. In 2017, Galappatthy and colleagues published an article in the International Journal of Rheumatic Diseases, analyzing pregnancy outcomes and contraceptive methods in women with SLE and rheumatoid arthritis (RA). They found that patients with SLE had more pregnancies than those with RA, highlighting the need for greater attention to unintended pregnancies and adverse outcomes in SLE [8].

In 2011, Yazdany and colleagues found that most women with SLE who were at risk lacked recent contraceptive counseling. This study and several others underscored the importance of raising awareness about effective contraceptive measures in these women to eliminate pregnancy-related risks [9,10]. However, studies assessing contraceptive use and counseling among women with SLE during pregnancy are lacking globally. In 2013, Kittisiam and colleagues found that only one-third of women with SLE used effective contraceptives [11-13].

A diverse selection of contraceptive options is accessible to women in Saudi Arabia through licensed pharmacies and healthcare providers. The most common method is combined oral contraceptives, used by approximately 31.8% of women, followed by intrauterine devices (IUDs) at around 21%. Other frequently used options include the withdrawal method (16.4%) and male condoms (13.6%). However, studies encourage consideration of long-term contraception, such as IUDs, particularly for adolescents with lupus [14,15].

Despite recommendations supporting universal contraceptive counseling and proactive pregnancy prevention for women with SLE, a significant gap remains in clinical practice. Inadequate counseling, inconsistent contraceptive use, and limited patient awareness contribute to preventable high-risk pregnancies in this group. This is particularly concerning in regions with high SLE prevalence and rapidly evolving healthcare infrastructures, such as Saudi Arabia, where cultural, social, and systemic factors may influence reproductive health behaviors.

This study aimed to assess contraceptive utilization and counseling practices among Saudi women with SLE who are prescribed potentially teratogenic medications. In addition to estimating prevalence and patterns of contraceptive use, the study examined associations between contraceptive behaviors and demographic and clinical characteristics, including educational level, parity, disease duration, and pregnancy risk status. By identifying gaps in current practice and highlighting unmet needs, this research sought to inform strategies for improved reproductive healthcare delivery and promote better health outcomes for women living with SLE.

## Materials and methods

Study design

This was a cross-sectional study that assessed pregnancy risks and contraceptive practices among reproductive-aged women with SLE. Data were collected through a dual-method approach: a retrospective review of electronic health records (EHRs) and prospective structured telephone surveys conducted between December 2024 and March 2025.

Study population and sampling

The study population consisted of women aged 18 to 45 years with a confirmed diagnosis of SLE identified from the EHR systems of King Saud University Medical City, a tertiary hospital in Riyadh, Saudi Arabia. Women who were postmenopausal or had undergone hysterectomy were not excluded; however, they were categorized as not at risk for pregnancy for the purposes of pregnancy-risk and contraceptive analyses. A convenience sampling method was employed, whereby all eligible patients meeting the inclusion criteria during the study period were invited to participate. This sampling method was selected because the clinic's patient flow was unpredictable, and not all patients visiting during the study period met the strict inclusion criteria.

During the study period, all eligible women identified through the EHR system were approached for participation. Due to the retrospective nature of patient identification and logistical constraints of telephone-based recruitment, precise documentation of unreachable or declining participants was not systematically recorded. However, the final sample included 399 women who consented to and completed the survey.

Participants were recruited using convenience sampling from a single tertiary care center; therefore, the reported prevalence reflects institutional prevalence and may not be representative of the broader population.

Data collection

Data were obtained from two primary sources: EHRs, which were used to extract clinical data, including diagnosis, medications (specifically prescriptions for azathioprine or cyclophosphamide), and demographic details; and structured telephone surveys, which were administered to consenting participants to gather detailed information on pregnancy risk, contraceptive practices, and other patient-reported outcomes. All telephone interviews were conducted by trained researchers using a standardized script to ensure consistency in question delivery and response recording. Interviewers underwent prior training to minimize variability, and no discretionary modifications to the survey questions were permitted.

Patient and public involvement

Patients or the public were not involved in the design, conduct, reporting, or dissemination of this research. This approach was justified by the study's design, which combined a retrospective EHR review with time-limited survey-based data collection, making early-stage patient involvement logistically unfeasible.

Instruments used

The questionnaire was reviewed and approved by the Research Center at King Saud University and was available in both Arabic and English. Participants completed the questionnaire in their preferred language. Consistent contraceptive use was operationally defined as self-reported use of a contraceptive method during every sexual encounter within the preceding three months. “Intermittent use” referred to inconsistent or occasional use during the same period, while “non-use” indicated no contraceptive use.

The instruments used in this study were based on previous studies [10]. Demographic characteristics (age, marital status, and education), clinical SLE data (disease duration, activity, and medications), and reproductive histories (pregnancy outcomes and abortions) were retrieved from the EHR system. Subsequently, trained researchers conducted standardized telephone surveys with eligible women to assess pregnancy risk, contraceptive practices (methods and consistency of use), and receipt of contraceptive counseling.

Disease activity and lupus flares were assessed using the Systemic Lupus Activity Questionnaire (SLAQ) scoring system, which is recognized as a reliable tool for patient assessment and evaluation [16,17].

Survey responses were cross-verified with EHR documentation when available to enhance data reliability. When discrepancies arose between EHR documentation and patient-reported responses, a predefined hierarchy was applied. Objective clinical variables (e.g., diagnosis, medication prescriptions, and laboratory results) defaulted to EHR records. Behavioral variables and adherence-related measures (e.g., contraceptive use frequency and counseling recall) were retained as patient self-reports, as these could not be reliably inferred from documentation alone.

The EHR system used was e-SiHi, an electronic health information system that contains various patient-level variables, including demographics, diagnoses, problem lists, medications, vital signs, and laboratory data.

Statistical analysis

The data were analyzed using IBM SPSS Statistics for Windows (Version 28.0; IBM Corp., Armonk, NY, USA, 2021). Descriptive statistics were used to summarize the data. Categorical variables were reported as frequencies and percentages, while continuous variables were expressed as mean±SD. Associations between categorical variables were assessed using the chi-square test, with Fisher’s exact test applied when expected cell counts were <5. A two-tailed significance threshold of p<0.05 was used for all tests. The analysis was strictly descriptive and associative, and no additional inferential tests were performed.

Sample size estimation

The required sample size was calculated a priori using the formula for single population proportions: n=Z2×p(1-p)/c2. Based on this calculation, a minimum sample size of 384 participants was required. All adult female SLE patients who agreed to participate were included. In this formula, n represents the required sample size; Z is the Z-statistic corresponding to the desired confidence level (1.96 for a 95% CI); p is the estimated proportion (0.50 was used to maximize the sample size, as no prior local prevalence data were available); and c is the desired margin of error (set at 0.05).

Ethical considerations

This study was approved by the Institutional Review Board (IRB) under approval number 24/1839/IRB. Verbal consent was obtained from participants during telephone surveys. The data collector ensured that participation in the study was voluntary and assured participants that their responses would not affect the quality of care they receive at the hospital.

## Results

The study population (n=399) was predominantly composed of married women (96.5%, n=385), with a small proportion of unmarried participants (3.5%, n=14). Educational attainment varied across the cohort; the largest group had a college-level education or higher (40.9%, n=163), followed by women who could read and write (33.8%, n=135) and high school graduates (25.3%, n=101). The mean disease duration was 14.8±7.3 years.

Most participants reported no history of hysterectomy (90%, n=359), while 10% (n=40) had undergone the procedure. Pregnancy history demonstrated a wide range of parity, with most participants reporting three to four pregnancies (15.5%-21.3%). A minority of women (8.8%, n=35) had never been pregnant, and extreme multiparity (>5 pregnancies) was uncommon, with only one participant reporting 14 pregnancies (Table 1).

**Table 1:** Demographics and clinical data

	N=339
Marital status	
Single	14 (3.35%)
Married	385 (96.5%)
Educational level	
Read and write	135 (33.8%)
High school	101 (25.3%)
College and above	163 (40.9%)
Disease duration in years (mean±SD)	14.8±7.3
History of hysterectomy	
Yes	40 (10%)
No	359 (90%)
Number of pregnancies	
Never	35 (8.8%)
1	20 (5%)
2	38 (9.5%)
3	62 (15.5%)
4	85 (21.3%)
5	65 (16.3%)
6	49 (12.3%)
7	12 (3%)
8	25 (6.3%)
9	6 (1.5%)
14	1 (0.3%)

Regarding disease activity, most participants reported moderate lupus flares during the preceding three months (56.4%, n=225), followed by mild flares (40.4%, n=161). Severe flares were rare (1.5%, n=6), while only 1.8% (n=7) reported no flares, indicating that active disease, predominantly of moderate severity, was common in this cohort (Table 1).

The reproductive history of participants revealed that 84.2% (n=336) had live births, while pregnancy losses included early miscarriages (13.5%, n=54), late miscarriages or stillbirths (17%, n=68), and ectopic pregnancies (1%, n=4). Additionally, 21.1% (n=84) reported a history of induced abortion, indicating varied pregnancy outcomes in the study population (Table 2).

**Table 2:** Reproductive history of the study participants ^*^Non-mutually exclusive

Pregnancy outcomes	N=363^*^
Live births	336 (84.2%)
Early miscarriages	54 (13.5%)
Ectopic pregnancies	4 (1%)
Late miscarriages/stillbirths	68 (17%)
Induced abortions	84 (21.1%)

Among women aged <45 years, 225 were prescribed potentially teratogenic medications (azathioprine or cyclophosphamide). A highly significant association was observed between pregnancy-risk status and teratogenic medication use (χ²(1)=12.89, p<0.001). Women who were postmenopausal or who reported no pregnancy risk (postmenopausal or no male partner, including prior hysterectomy) were classified as not at risk for pregnancy and analyzed separately. Notably, all women in this group were prescribed teratogenic medications (100%, n=16/16), compared with 54.6% of women at risk for pregnancy (n=209/383). No statistically significant association was found between receipt of contraceptive counseling at the time of diagnosis among women at risk for unintended pregnancy and teratogenic medication use (Pearson χ²(1)=2.64, p=0.104). In contrast, a strong association was identified between contraceptive use frequency and teratogenic medication prescription (χ²(2)=127.18, p<0.001); women who never used contraceptives had a prescription rate of 90.1%, compared with 64.2% among intermittent users (“sometimes”) and only 17.2% among consistent users (p<0.001). Additionally, significantly fewer women who reported exclusively positive responses regarding contraceptive use reported receiving contraceptive counseling in the past year (38.7%, n=87) compared with other respondents (80.7%, n=138), with a statistically significant difference (p<0.001) (Table 3). The higher prevalence of contraceptive use compared with reported counseling suggests that some participants initiated or continued contraception independently or based on counseling received outside the assessed timeframe, without recent documented contraceptive counseling within lupus care (Table 3).

**Table 3:** Pregnancy risk status, contraceptive use, and counseling among women aged <45 years receiving potentially teratogenic medications IUD: intrauterine device

Variable	Yes, n (%)	No, n (%)	Chi-square (df)	P-value
Not at risk for pregnancy (postmenopausal/no male partner)	16 (100.0)	209 (54.6)	χ²(1)=12.89	<0.001
At risk for pregnancy	209 (54.6)	174 (45.4)	χ²(1)=12.89	<0.001
Received contraceptive counseling at diagnosis: yes	207 (55.3)	18 (72.0)	χ²(1)=2.64	0.104
Received contraceptive counseling at diagnosis: no	18 (4.7)	167 (27.0)	χ²(1)=2.64	0.104
Received contraceptive counseling in past year: yes	87 (38.7)	141 (61.3)	χ²(1)=71.92	<0.001
Received contraceptive counseling in past year: no	138 (61.3)	33 (19.0)	χ²(1)=71.92	<0.001
Frequency of contraceptive use: never	91 (40.4)	10 (5.7)	χ²(2)=127.18	<0.001
Frequency of contraceptive use: sometimes	113 (50.2)	63 (36.2)	χ²(2)=127.18	<0.001
Frequency of contraceptive use: always	21 (9.3)	101 (58.0)	χ²(2)=127.18	<0.001
Contraceptive method: none	91 (40.4)	56 (32.2)	χ²(2)=100.57	<0.001
Contraceptive method: barrier	39 (17.3)	108 (62.1)	χ²(2)=100.57	<0.001
Contraceptive method: IUD	87 (38.7)	10 (5.7)	χ²(2)=100.57	<0.001

A significant association was observed between educational level and receipt of contraceptive counseling in the past year (χ²(2)=12.36, p=0.002), with counseling rates varying across education groups. Women with a high school education reported the highest rate of counseling (70.3%), followed by women with basic literacy (57.8%), while college-educated women had the lowest rate (48.5%). Counseling at the time of diagnosis also differed significantly by educational level (χ²(2)=12.17, p<0.001), with 100% of women with basic literacy (read and write) reporting receipt of counseling.

Educational level was strongly associated with contraceptive use frequency in the preceding three months (χ²(4)=142.97, p<0.001). Non-use of contraception was absent among women with basic literacy, with the majority reporting consistent use (57.8%). In contrast, nearly half of college-educated women (48.5%) reported no contraceptive use.

Contraceptive method choice also differed significantly by educational level (χ²(4)=108.82, p<0.001), with IUD use observed exclusively among college-educated women (46.6%), while women with basic literacy reported only barrier methods or no contraception.

Disease duration differed significantly across contraceptive use frequency groups (F=38.88, p<0.001) and contraceptive method groups (F=27.13, p<0.001). Barrier-method users had the longest mean disease duration (17.14±6.34 years), followed by non-users (15.30±8.06 years), whereas IUD users had the shortest duration (10.61±5.34 years).

A significant association was observed between contraceptive use frequency and number of pregnancies (χ²(2)=24.20, p<0.001) (Tables 4, 5).

**Table 4:** Counseling outcomes by participant characteristics ^*^Calculated using the independent-samples t-test for continuous variables.

Characteristics	Counseling at diagnosis, n (%)	χ² (df)	P-value	Counseling in the past year, n (%)	χ² (df)	P-value
Number of pregnancies <5	220 (91.3)	χ²(1)=6.21	0.018	163 (67.6)	χ²(1)=18.47	<0.001
Number of pregnancies ≥5	154 (97.5)	χ²(1)=6.21	0.018	65 (41.1)	χ²(1)=18.47	<0.001
Educational level: read and write	135 (100.0)	χ²(2)=12.17	<0.001	78 (57.8)	χ²(2)=12.36	0.002
Educational level: high school	92 (91.1)	χ²(2)=12.17	<0.001	71 (70.3)	χ²(2)=12.36	0.002
Educational level: college and above	147 (90.2)	χ²(2)=12.17	<0.001	79 (48.5)	χ²(2)=12.36	0.002
Disease duration (years, mean±SD)	15.07±7.12	-	0.016^*^	14.87±7.00	-	0.927^*^

**Table 5:** Frequency of contraceptive use by number of pregnancies

Number of pregnancies	Never, n (%)	Sometimes, n (%)	Always, n (%)	χ² (df)	P-value
<5	48 (19.9)	98 (40.7)	95 (39.4)	χ²(2)=24.20	<0.001
≥5	53 (33.5)	78 (49.4)	27 (17.1)	χ²(2)=24.20	<0.001

A significant association was observed between the number of pregnancies and the contraceptive method used (χ²(2)=22.32, p<0.001) (Table 6). Women with fewer than five pregnancies were more likely to use barrier methods (45.6%) compared with women with five or more pregnancies (23.4%). Conversely, IUD use was nearly twice as common among women with five or more pregnancies (32.9%) compared with those with fewer pregnancies (18.7%). The number of pregnancies was also significantly associated with receipt of contraceptive counseling at the time of diagnosis (χ²(1)=6.21, p=0.018) and with receipt of counseling in the past year (χ²(1)=18.47, p<0.001). No significant difference in disease duration was observed between women who received counseling and those who did not (Table 6).

**Table 6:** Contraceptive method by number of pregnancies IUD: intrauterine device

Number of pregnancies	No method, n (%)	Barrier method, n (%)	IUD, n (%)	χ² (df)	P-value
<5	86 (35.7)	110 (45.6)	45 (18.7)	χ²(2)=22.32	<0.001
≥5	69 (43.7)	37 (23.4)	52 (32.9)	χ²(2)=22.32	<0.001

## Discussion

Our study highlighted that most participants received contraceptive counseling at the time of diagnosis. However, less than half received counseling in the past year, underscoring the lack of continuous education for women taking potentially teratogenic medications. These results are consistent with another study, which showed that contraceptive counseling was relatively inadequate [10,18].

Given the cross-sectional design of this study, the associations observed should not be interpreted as causal relationships. The findings represent correlations measured at a single time point and do not establish temporal directionality.

Overall contraceptive use among participants in the past three months was low, revealing deficiencies in reproductive risk mitigation among women with SLE, which may result in unintended pregnancies. The low compliance rates shown in this study were consistent with a previous study that demonstrated insufficient contraceptive use among patients with SLE [19]. Therefore, this study highlights the critical need for systematic pregnancy prevention protocols in this high-risk population taking potentially teratogenic medications.

A comparison between our findings and data from the general Saudi population reveals significant differences in both the prevalence and types of contraception used. While external studies have reported lower rates of IUD use, ranging from 5.7% to 33.2%, our study indicates that participants relied primarily on IUDs [20,21].

Similarly, the reported use of barrier methods, typically defined here as male condoms, was generally lower in our cohort than in most population-based studies. For instance, studies have reported condom use rates as high as 27.3%, whereas our findings reflect lower utilization [22,23]. However, another study reported a lower rate of 13.6% [14]. The inconsistency in these results may be explained by differences in sample sizes and the exclusion of males in this research work. These differences emphasize the unique reproductive health needs of Saudi women living with SLE and highlight the importance of tailored contraceptive counseling for this population.

Paradoxically, a strong association was observed between college-educated women and a lack of contraceptive use, while those with basic literacy reported exclusive barrier method use or no contraception. These results contradict findings from other studies showing that contraceptive use increases with educational level [24,25]. Therefore, the current findings may reflect greater reproductive autonomy among educated women, leading to intentional non-use. However, they also highlight how educational disparities may influence contraceptive decision-making, underscoring the need for targeted interventions to address inequities in contraceptive practices across educational groups.

The results also showed that women with longer disease duration demonstrated significantly higher rates of consistent contraceptive use, possibly reflecting the cumulative impact of SLE progression and treatment-related risks. This pattern aligns with established evidence that cumulative disease activity increases disease burden on women, such as accelerating organ damage and pushing them toward patient-initiated pregnancy avoidance due to their lived experience with the disease [26].

Disease duration also had a significant impact on contraceptive choice. Specifically, longer disease duration was associated with the use of barrier methods only. To our knowledge, this association has not been previously reported.

Nevertheless, this study provides valuable insights into contraceptive practices among Saudi women with SLE. The predominantly cross-sectional design limits causal interpretation of the observed associations. The demographic and clinical characteristics, including educational disparities, parity-based method selection, and disease duration trends, reflect real-world patterns but do not establish temporal relationships or underlying mechanisms. Therefore, longitudinal studies are needed to determine whether the identified factors (e.g., education level and disease duration) directly influence contraceptive behaviors or are mediated by unmeasured confounders such as socioeconomic status, healthcare access, or cultural norms. Finally, the lack of consistent contraceptive use, coupled with the prevalence of teratogenic prescriptions, suggests the need to establish an effective teratogenic risk-awareness protocol for patients with SLE.

Limitations

This study has several limitations. First, its cross-sectional design precludes causal inferences regarding the observed associations. Additionally, as a single-center study utilizing convenience sampling, the findings reflect prevalence within our institution and are subject to selection bias, thereby limiting external validity and generalizability to broader populations. The unique demographics and clinical practices of our institution may not represent other healthcare settings in Saudi Arabia.

Second, contraceptive use data were self-reported through telephone interviews, which may introduce recall bias. Furthermore, the absence of a standardized contraceptive counseling protocol across the cohort may have resulted in variability in counseling practices and outcome assessment.

Third, several potentially relevant variables were not included in the analysis, such as comorbidities, current medications, and detailed documentation of azathioprine and cyclophosphamide dosages and duration of exposure. In addition, multivariable regression analysis was not performed; therefore, observed associations between contraceptive behaviors and factors such as education level, parity, or disease duration may be influenced by residual confounding variables, including age, socioeconomic status, and healthcare access.

Finally, sociocultural determinants such as marital norms and patterns of sexual activity, which are particularly relevant in the Saudi context, were not directly measured. These contextual factors may substantially influence contraceptive decision-making.

Future multicenter studies incorporating broader populations, standardized counseling protocols, validated culturally adapted instruments, detailed medication exposure data, and adjusted multivariable analytical models are warranted to enhance generalizability and identify independent predictors of contraceptive use.

Recommendations

These findings underscore the need for longitudinal studies to determine whether the identified factors (e.g., education level and disease duration) directly influence contraceptive behaviors or whether they are mediated by unmeasured confounders such as socioeconomic status, healthcare access, or cultural norms. A prospective study should also investigate interventions to enhance patient-provider communication regarding teratogenic risks.

## Conclusions

This study highlights significant associations between education, parity, disease duration, and contraceptive behaviors, while recognizing that causal inferences cannot be established due to the cross-sectional design. Integrating these findings into clinical practice may help optimize reproductive care for women with SLE, reducing unintended pregnancies while minimizing teratogenic exposures. Our findings also demonstrate that contraceptive counseling at the time of diagnosis and during follow-up is limited, underscoring the critical need for continuous educational programs for women with SLE.
